# Molecular Dynamics Study of the Aggregation Behavior of *N*,*N*,*N*′,*N*′-Tetraoctyl Diglycolamide

**DOI:** 10.1021/acs.jpcb.2c02198

**Published:** 2022-08-17

**Authors:** Daniel Massey, Andrew Masters, Jonathan Macdonald-Taylor, David Woodhead, Robin Taylor

**Affiliations:** † Department of Chemical Engineering, The University of Manchester, Oxford Road, Manchester M13 9PL, U.K.; ‡ National Nuclear Laboratory, 5th Floor Chadwick House, Warrington Road, Birchwood Park, Warrington WA3 6AE, U.K.; § National Nuclear Laboratory, Central Laboratory, Sellafield, Seascale CA20 1PG, U.K.

## Abstract

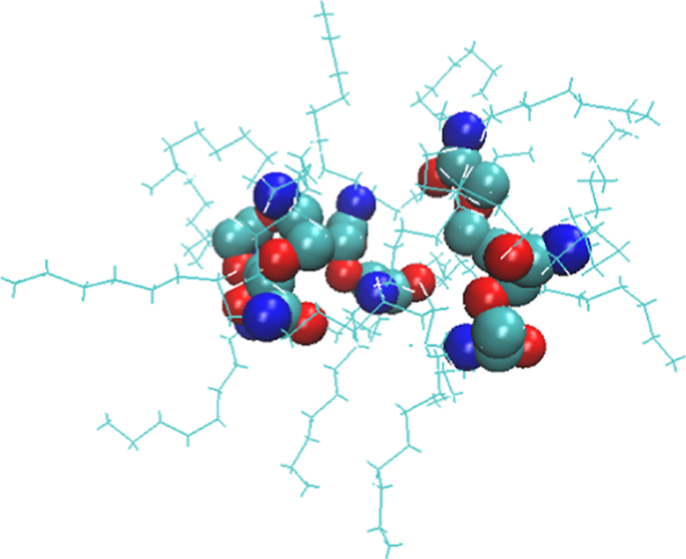

Liquid–liquid extraction is a commonly used technique to separate metals and is a process that has particular relevance to the nuclear industry. There has been a drive to use environmentally friendly ligands composed only of carbon, hydrogen, nitrogen, and oxygen. One example is the i-SANEX process that has been developed to separate minor actinides from spent nuclear fuel. The underlying science of such processes, is, however, both complex and intriguing. Recent research indicates that the liquid phases involved are frequently structured fluids with a hierarchical organization of aggregates. Effective flow-sheet modeling of such processes is likely to benefit from the knowledge of the fundamental properties of these phases. As a stepping stone toward this, we have performed molecular dynamics simulations on a metal free i-SANEX system composed of the ligand *N*,*N*,*N*′,*N*′-tetraoctyl diglycolamide (TODGA), diluent hydrogenated tetrapropylene (TPH), and polar species water and nitric acid. We have also studied the effects of adding *n*-octanol and swapping TPH for *n*-dodecane. It would seem sensible to understand this simpler system before introducing metal complexes. Such an understanding would ideally arise from studying the system’s properties over a wide range of compositions. The large number of components, however, precludes a comprehensive scan of compositions, so we have chosen to study a fixed concentration of TODGA while varying the concentrations of water and nitric acid over a substantial range. Reverse aggregates are observed, with polar species in the interior in contact with the polar portions of the TODGA molecules and the organic diluent on the exterior in contact with the TODGA alkyl chains. These aggregates are irregular in shape and grow in size as the amount of water and nitric acid increases. At a sufficiently high polar content, a single extended cluster forms corresponding to the third phase formation. No well-defined bonding motifs were observed between the polar species and TODGA. The cluster size distribution fits an isodesmic model, where the Gibbs energy change of adding a TODGA molecule to a cluster ranges between 4.5 and 7.0 kJ mol^–1^, depending on the system composition. The addition of *n*-octanol was found to reduce the degree of aggregation, with *n*-octanol acting as a co-surfactant. Exchanging the diluent TPH for *n*-dodecane also decreased the aggregation. We present evidence that this is due to the greater penetration of *n*-dodecane into the reverse aggregates. It is known, however, that the propensity for the third phase formation is greater with *n*-dodecane as the diluent than is the case with TPH, but we argue that these two results are not contradictory. This research casts light on the driving forces for aggregation, informs process engineers as to what species are present, and indicates that flow-sheet liquid–liquid extraction modeling might benefit by incorporating an isodesmic aggregation approach.

## Introduction

The most common way to separate metals is liquid–liquid extraction.^1−8^ Taking nuclear fuel recycling as an important example, spent nuclear fuel is delay-stored to reduce the effects of short-lived radioactive species and is then dissolved in nitric acid. This is contacted with an organic diluent and an appropriate ligand. The ligand binds selectively to the desired metal or metals to form a complex, and this complex moves from the aqueous to the organic phase, thus achieving the separation. The plutonium–uranium reduction extraction (PUREX) process for extracting uranium and plutonium was patented in 1947 and is still in common use.^9,10^ The ligand is *n*-tributyl phosphate (TBP) and sophisticated flow-sheet models exist for this process.^11−14^ More recently, other extraction processes, based on more environmentally friendly CHON ingredients, containing only C, H, O, and N, are coming to the fore.^15,16^ These can be decomposed, yielding only gaseous products, thereby reducing the volume of radioactive waste.

What is becoming increasingly clear, however, is that the organic phase, containing the extracted metal, is far from a simple solution. Both atomistic computer simulations, small-angle neutron (SANS) and X-ray (SAXS) scattering experiments, indicate the presence of discrete aggregated structures, resulting from a complex interplay between the interactions between metal complexes and the reverse-emulsion-like structures resulting from having an amphiphilic ligand in a non-polar environment.^1,7,17−26^ At sufficiently high metal loadings or at sufficiently high acidities, these discrete aggregates within the organic phase fuse either to form a precipitate or else to form what appears to be a continuous, percolating structure containing connected regions of polar molecules, such as water and nitric acid, and connected regions of non-polar molecules, such as the hydrocarbon diluent. The metal complexes are in the polar region, while unbound amphiphilic ligands are largely at the interface. This is the so-called third phase, which, while very interesting, is highly undesirable in any extraction process, not least because high concentrations of uranium and plutonium may risk nuclear criticality.^1,19,20,22,27−37^

The detailed structures of these phases and the molecular driving forces for the phase transition to the third phase are still far from clear. Molecular dynamics simulations struggle with achieving a large enough system size to capture all the details of the aggregated structures, with the third phase being an especially difficult challenge. SANS and SAXS experiments require the use of models to interpret the scattering profiles and simplifying assumptions concerning the shapes of the aggregates have to be made. Arguably, a combination of atomistic computer simulation and SANS/SAXS experiments is a powerful way to proceed, with the experimental work providing at least partial validation of the simulations and the simulations giving detailed information on the molecular interactions and structures. What seems to emerge from these studies is that the aggregates are not simple, ordered structures. They are not mono-disperse and do not have a simple shape. Snapshots from the simulation indicate very disordered, flexible structures rather than the spheres, cylinders, and bi-layers that form the framework for understanding amphiphiles in aqueous solution.^38,39^

**Figure 1:**
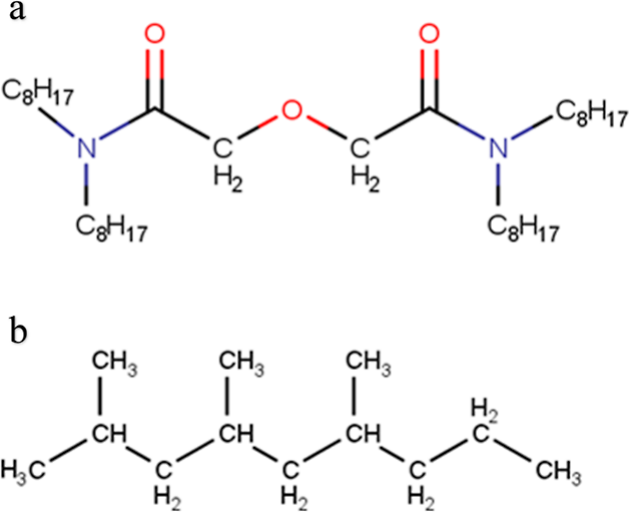
(a) Structure of a TODGA molecule. (b) Structure of TPH.

In this paper, we present results aimed at increasing our understanding of these structured organic phases. We consider, as a ligand, the diglycolamide *N*,*N*,*N*′,*N*′-tetraoctyl diglycolamide, commonly known as TODGA. Its chemical structure is given in Figure 1a^40^ drawn using the software Marvin 18.3.0, 2018, ChemAxon.^41^ TODGA is a key ingredient in the i-SANEX process,^42−47^ which shows great promise for the separation of minor trivalent actinides (e.g. americium and curium) from high-level waste (HLW) solutions generated by PUREX or similar extraction processes. The removal of actinides significantly reduces the time taken for nuclear waste to be isolated from the environment before its radiotoxicity falls below that of the uranium ore. Also, the minor actinides are the main contributors to heat generation in the longer term (once plutonium is separated from spent fuel), and their removal enables a more compact arrangement of radioactive waste in the disposal facility, thus reducing the size of the disposal facility. This is the so-called partitioning and transmutation strategy for recycling actinides in a fully closed fuel cycle.^48−52^ In this strategy, after the PUREX process has been used to extract uranium, plutonium, and possibly other metals such as neptunium and technetium, the remaining HLW is contacted with a mixture of a diluent hydrocarbon, TODGA, and *n*-octanol. A commonly used diluent is hydrogenated tetrapropylene (TPH). This is a branched dodecane and its structure is shown in Figure 1b.^53^ Other diluents are also used, for example, isane and odorless kerosene, of which *n*-dodecane is a major constituent. Furthermore, the addition of *n*-octanol as a phase modifier has been shown to reduce the propensity for third phase formation and/or precipitation.^8,23,33,54−56^

The i-SANEX organic phase is a complex mixture of a metal complex, TPH, TODGA, *n*-octanol, water, and nitric acid. In order to study this system, it makes sense to build up complexity in stages. We thus do not consider the metal in this article but focus instead on the properties of the metal-free mixture, which is still a complicated system. That said, the aggregates formed in the metal-free system may be regarded as receptacles for metal complexes, so this study also provides some useful background for the analysis of metal-containing systems.^40^ This metal-free system has been studied experimentally using, among other techniques, SAXS and SANS measurements, both in a normal organic phase and in the third phase. These studies indicate that at low acidity and water concentrations, TODGA in the organic phase will mainly be found in the form of monomers and dimers, but at higher acid and water concentrations, small reverse-micelles appear containing a small number of TODGA monomers, typically in the range of 3–6, along with associated acid and water.^17,19,21,57^

Our aim in this article is to study the metal-free i-SANEX system, exploring the link between molecular interactions and the aggregates that result. Previous simulations of metal-free systems have shown that diglycolamides form reverse structures in a non-polar diluent, and there has been extensive analysis of the molecular organization of these clusters and the distributions of cluster sizes and compositions.^19,21,58−63^ A very recent article reports the results of large-scale simulations of the TODGA/*n*-dodecane/water/nitric acid system as well as providing a detailed description of the molecular structure of the aggregates.^64^

In this article, we aim to add to this existing knowledge by looking at the thermodynamics of aggregate formation and studying the effects of changing the diluent and of adding *n*-octanol. Yaita et al.^18^ and Nave et al.^19^ report experimental results on these systems, including scattering experiments (SANS and SAXS) that provide information on clustering. They also provide the full composition of the organic phase, including the concentration of water. As shown in a recent study, water plays an important role in tuning the lanthanide selectivity of diglycolamides.^65^ It is thus very likely that it also plays a significant role in actinides. A knowledge of its concentration can therefore be invaluable when comparing simulation with experiment. Yaita et al.^18^ studied a metal-free organic phase where the TODGA and nitric acid concentrations in the organic phase were 0.1 M and 0.05 M, respectively. Nave et al.^19^ focused on an organic phase with the same TODGA concentration as above but a nitric acid concentration of 0.01 M. In both cases, these organic phases were in equilibrium with corresponding aqueous phases. These systems have been previously simulated^63,64^ and the clustering discussed. Bell et al.^61^ studied nitric acid extraction into TODGA at a higher nitric acid concentration. For 0.1 M TODGA, their data go up to an organic nitric acid concentration of 0.132 M. They also present modeling that suggests that four water molecules are extracted with each nitric acid molecule. It is of interest to study these higher concentrations where aggregation effects are more pronounced. This is both because of the intrinsic scientific interest of such systems and also because such studies may help inform the flow-sheet modeling used in practical applications. Such simulation studies have been reported previously^63,64^ and one of our aims here is to extend the composition space investigated. After looking briefly at pure TODGA and TODGA/TPH mixtures, we investigate the effects of varying the amount of water and nitric acid in the system on local structure and aggregate formation. We then add *n*-octanol to study its effect on cluster formation. We also briefly consider the effects of changing the diluent from TPH to *n*-dodecane. We finish up with a discussion.

## Methodology

The OPLS-2005 all-atom forcefield and combining rule^66^ was used for TPH, *n*-dodecane, and *n*-octanol and also for the non-bonded parameters in TODGA. The TIP3P model was used for water for compatibility with this forcefield. The TODGA atomic charges were those previously reported by Singh et al.^63^ The nitric acid parameters came from Price et al.^67^ (also using geometric combining following the OPLS procedures) and are given explicitly in the Supporting Information. This nitric acid model was also used by Mu et al.^22^ in their study of a TBP system. It should be noted that this forcefield does not allow for the breakage and formation of bonds, which means that there is no mechanism allowing nitric acid to dissociate into hydronium and nitrate ions and no mechanism allowing for the protonation of the carboxyl oxygens in TODGA. For this, one requires more sophisticated simulation techniques, such as constant pH simulations. Recent examples of such studies are given in refs (68) and (69). Experimental studies by Musikas and Hubert^70^ on the related ligand *N*,*N*′-dimethyldioctyl malonamide indicate that in the presence of nitric acid, the protonated ligand is extracted in relatively low concentrations, estimated to be at a mole fraction of 0.075 of the total ligand extracted under the conditions studied. Lefrançois et al.^71^ also discuss such effects based on an analysis of NMR data, focusing on the extraction of a quaternary malonamide. Their studies indicate that protonation is a significant effect in the third phase but less so in the organic phase. Singh et al.^63^ ran simulations on the i-SANEX system considered here, both with molecular and dissociated nitric acid in the organic phase, finding little effect on the degree of clustering. We are thus hopeful that our neglect of protonation will not seriously affect our conclusions.

The simulations were completed using the GROMACS 2018.4 software package.^72−79^ All simulations used cubic periodic boundary conditions and a time step of 1 fs. For van der Waals and short-range Coulomb interactions, a 1.2 nm cut-off was implemented and potential shift functions were applied from 0.9 to 1.2 nm to conserve energies at the cut-off. Particle-mesh Ewald summation^80^ was used for the long-range electrostatic potential. The Fourier spacing was 0.12 nm^–1^, and cubic interpolation was employed. Bonds involving hydrogen were constrained using LINCS. Simulations were all performed in the isobaric isothermal *NpT* ensemble using a leapfrog Verlet integrator at a pressure and a temperature of 1 bar and 298.15 K, respectively. For each system configuration, the molecules were randomly inserted into a 6 nm cubic box. The systems were equilibrated for 10 ns, first conducting a 5 ns run using the velocity-rescaling thermostat and the Berendsen barostat with a time constant of 1 ps, followed by a second run using the Nosé–Hoover thermostat and the Parrinello–Rahman barostat with a time constant of 5 ps. The production runs were for 40 ns, again using the Nosé–Hoover thermostat and the Parrinello–Rahman barostat.

To help analyze the local structure of the systems, we make use of co-ordination numbers, CN_αβ_, defined by
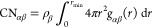
1

Here, ρ_β_ is the number density of atoms of type β and *g*_αβ_(*r*) is the atom–atom radial distribution function for atom types α and β. *r*_min_ is the position of the first minimum of *g*_αβ_(*r*). This co-ordination number gives an estimate of the number of β atoms that are in the first co-ordination shell of an *α* atom.

In order to analyze the degree of aggregation, we made use of cluster analysis. This requires a criterion to decide whether two molecules are connected. GROMACS provides an in-built cluster analyzer where the connectivity criterion depends on the distance between two specified atoms. We are also interested in analyzing hydrogen-bonded clusters. For this, we used the algorithm of Sevick et al.,^81^ as modified by Mu et al.,^22^ to estimate the number and sizes of clusters formed by TODGA, HNO_3_, and H_2_O. The criterion for a hydrogen bond was a donor–acceptor distance of less than 0.36 nm and a bond angle between the donor, the hydrogen atom, and the atom bonded to the hydrogen of 150° or greater. We found that these criteria gave predictions in reasonable agreement with an intuitive inspection of simulation snapshots and that our general conclusions did not depend sensitively upon the precise numbers chosen.

## Results

### Pure TODGA System

A pure TODGA system containing 800 molecules was simulated at 1 bar and 298.15 K as described previously, except that a production run of 20 ns was found to be sufficient. The average density was 0.9373 ± 0.0003 g cm^–3^ as opposed to experimental values for pure TODGA of 0.910 g cm^–3^.^82^

The system size dependence of this result was found to be negligible. No doubt, the forcefield could be adjusted to give closer agreement with the experimental density, but considerably more experimental data are required to do such a fit reliably. As the purpose of this paper is to explore trends rather than to obtain quantitative predict ions, we believed this level of agreement to be acceptable.

### TODGA/TPH

**Figure 2:**
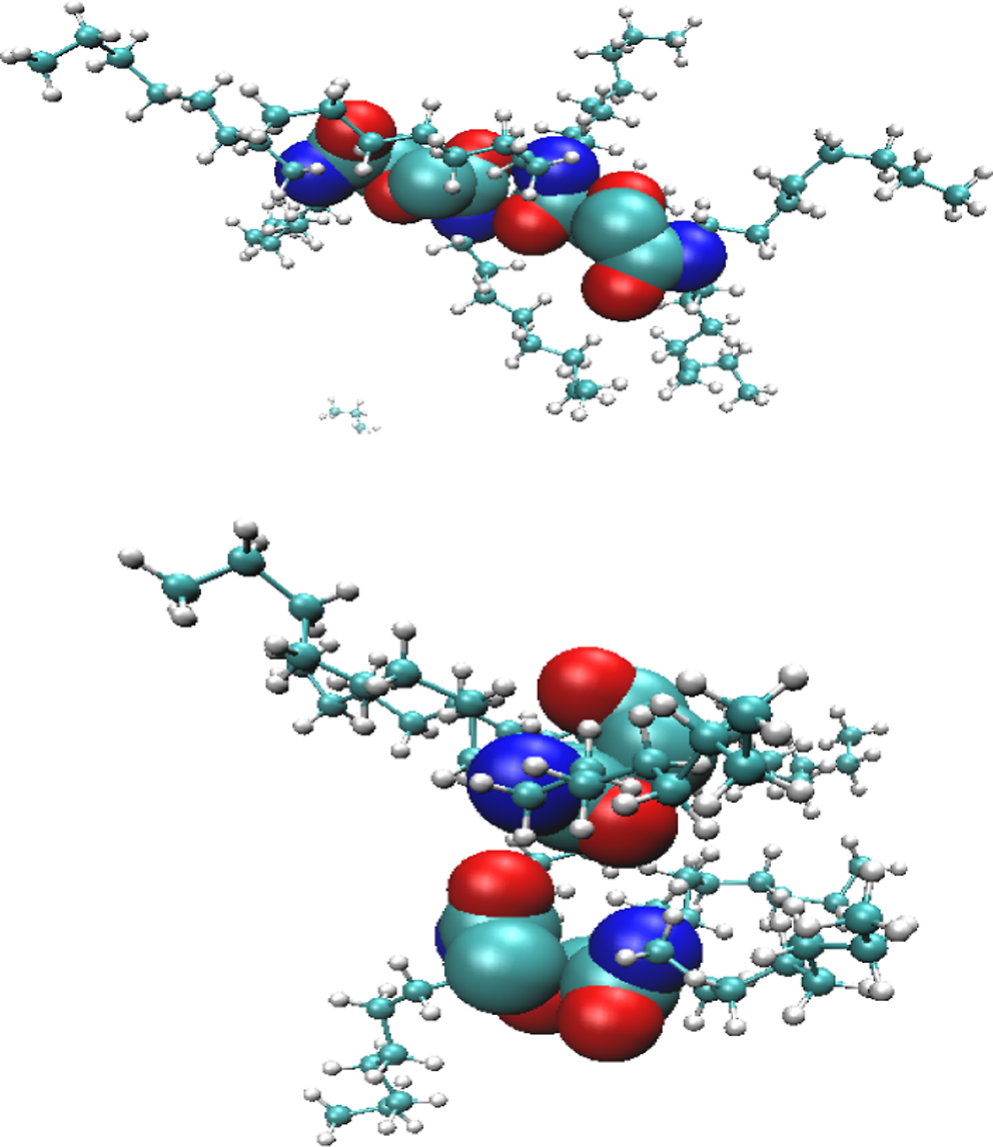
Snapshots of TODGA forming small clusters. Red—oxygen, blue—nitrogen, cyan—carbon, white—hydrogen.

**Figure 3:**
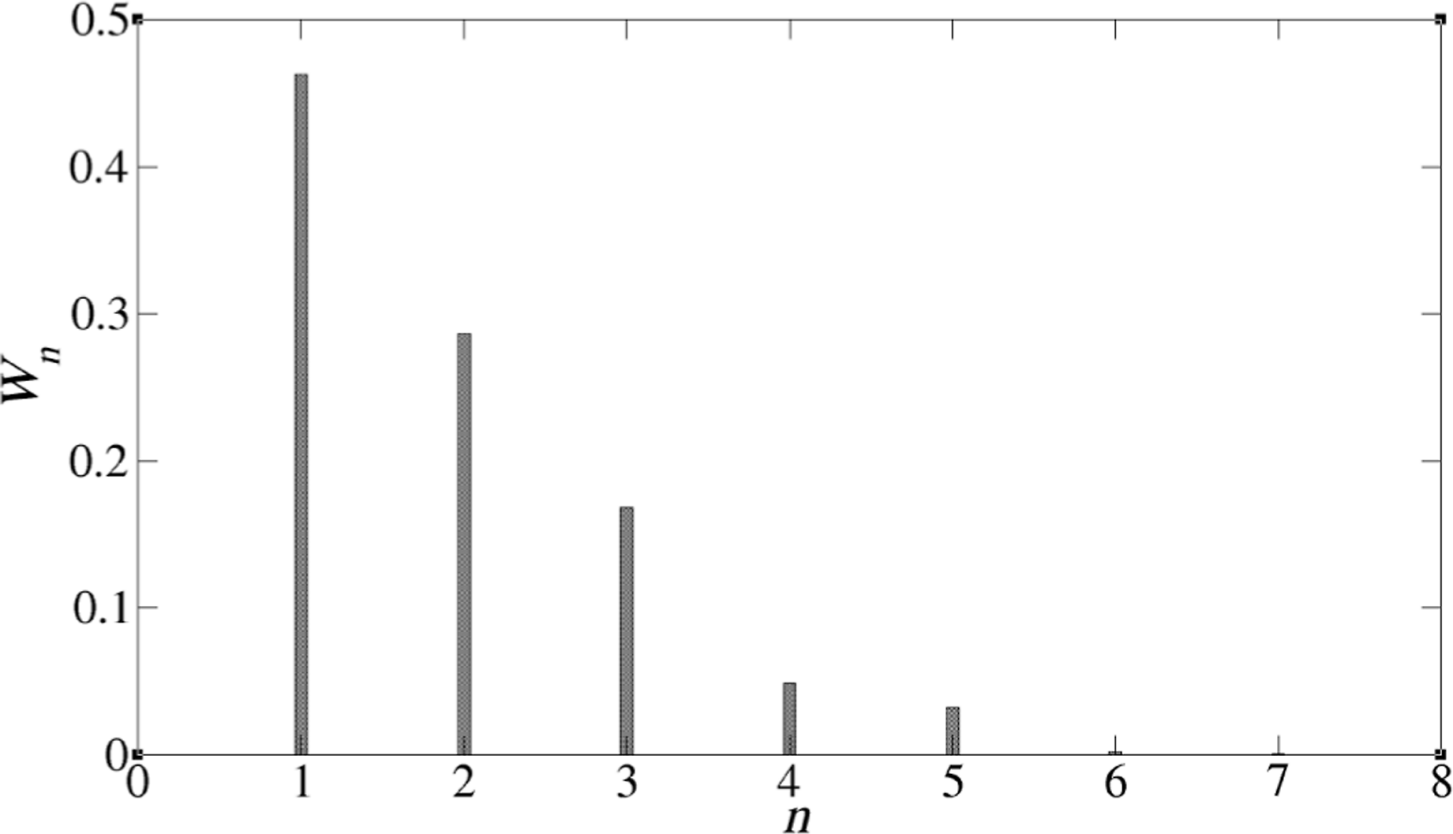
Cluster size distribution of TODGA in the TODGA-TPH system, defined in Table 1. Here, *n* is the number of TODGAs in a cluster, while *W*_*n*_ is the average mole fraction of TODGA in an *n*-cluster.

In the TODGA/TPH system, the composition was chosen to give a TODGA molarity of approximately 0.1, which is typically used in studies of this sort.^11,18,33,56,60,63,83−85^ This system comprised 10 TODGA molecules and 400 TPH molecules. The co-ordination number from the carbonyl oxygen–carbonyl oxygen distribution function was 0.8 while the ether oxygen–ether oxygen co-ordination number was 0.7. These numbers indicate an attraction between TODGA molecules. Visual inspection of simulation snapshots showed the formation of small clusters. Typical examples are shown in Figure 2. For a more detailed analysis of the molecular organization within these clusters, we refer elsewhere.^64^ These clusters are mobile, as witnessed by the very different molecular arrangements shown in the two snapshots in Figure 2. This variation indicates that the interactions between the polar groups are somewhat unspecific. We used the Gromacs^86^ cluster analyzer, where the connection criterion was a separation of less than 0.7 nm between two TODGA carbonyl oxygens. This distance corresponds to the first minimum of the distribution function. The analysis is an average over the 40 ns production run. A graph showing the cluster distribution is shown in Figure 3, indicating that the TODGAs exist mainly as monomers, dimers, or trimers. Here, *W*_*n*_ is the average mole fraction of TODGA molecules to be found in a TODGA cluster with aggregation number *n*.

### Effects of Acid and Water

We have systematically varied the amount of water and nitric acid in the organic phase to study how these polar molecules affect the liquid structure. Systems 1–3 have a water/nitric acid ratio of 2, while systems 4–6 have a water/nitric acid ratio of 4. For both compositions, we vary the amount of polar material, with details given in Table 1. The maximum nitric acid concentration studied was 0.11 M, while the largest organic nitric acid concentration reported by Bell et al.^61^ for a TODGA concentration of 0.1 M was 0.132 M. We thus believe our chosen compositions to be physically reasonable and not prone to phase separation. We carefully checked all our simulation runs for any signs of liquid–liquid demixing, and no such trends were observed. On the timescale of the simulation, extensive visual inspection indicated that molecules entered and left clusters on a timescale of approximately 1 ns, while the timescale for the formation or break-up of a whole cluster was approximately 3–5 ns. In Figure S6 in the Supporting Information, we show a plot of the TODGA cluster distribution over time for system 6, which has the highest content of water and nitric acid simulated. The plot shows no sign of cluster growth with time. These plots were similar for all systems studied. We thus believe that all the results reported here are for thermodynamically stable phases. Clustering is observed in all these systems. These clusters are irregular in shape and are dynamic in nature. It would be of interest to quantitatively compare this dynamics with experimental observations. Such studies have been conducted on micelles in aqueous phases, for example, as done by Aniansson et al.^87^ and would be an interesting future line of study.

**Table 1:** Compositions of Simulated Systems in Terms of Both Molarity, mol dm^–3^, and below the Number of Molecules Simulated^a^

system	1	2	3	4	5	6	third phase
TODGA	0.09	0.09	0.09	0.09	0.09	0.09	0.15
	10	10	10	10	10	10	200
H_2_O	0.07	0.15	0.23	0.15	0.30	0.45	0.07
	8	16	24	16	32	48	90
HNO_3_	0.04	0.08	0.11	0.04	0.07	0.11	0.77
	4	8	12	4	8	12	1000
TPH	400	400	400	400	400	400	5000

a Thus, for example, in system 1, the TODGA molarity was 0.09 mol dm^–3^ and 10 TODGA molecules were simulated.

We begin by looking at the interactions between the polar species and TODGA by making use of a co-ordination number analysis. A sub-set of results is shown in Table 2.

**Table 2:** Co-ordination Numbers for Certain Atoms in the Systems Simulated^a^

pair (first atom reference)	system 1	system 2	system 3	system 4	system 5	system 6	*r*_min_/nm
TODGA(O)–TODGA(O)	0.96	0.70	0.67	0.88	0.73	0.60	0.61
TODGA(O)–HNO_3_(H)	0.12	0.30	0.44	0.13	0.29	0.44	0.36
TODGA(O)–H_2_O(H)	0.21	0.28	0.31	0.37	0.48	0.46	0.25
HNO_3_(N)–HNO_3_(N)	0.04	0.11	0.19	0.06	0.12	0.20	0.68
HNO_3_(N)–H_2_O(O)	0.09	0.21	0.36	0.18	0.44	0.80	0.60
H_2_O(O)–H_2_O(O)	0.34	0.77	1.16	0.74	1.88	3.85	0.65

a TODGA(O) refers to a carbonyl oxygen atom in TODGA.

The polar region of TODGA corresponds to the two amide groups connected by an ether oxygen. Many of these atoms have significant partial charges (see the Supporting Information). It is therefore likely that all these atoms are involved, to a greater or lesser extent, in the formation of clusters via polar interactions. Analysis of the co-ordination numbers and the radial distribution functions indicate, however, that the prime binding site is the carbonyl oxygen. We thus begin by looking at which species bind to these carbonyl oxygens. The obvious candidates are hydrogen in nitric acid, hydrogens in water, and the positively charged atoms in TODGA, such as the carbonyl carbon. As noted previously, however, there only appear to be rather unspecific TODGA–TODGA interactions, so we focus here on the water and nitric acid interactions.

The data indicate that as the amount of polar material increases at a fixed water/nitric acid ratio, the nitric acid tends to displace water as the main co-ordinator to the carbonyl oxygen, implying that nitric acid has a stronger interaction than does water. This is the same trend as observed in TBP systems, but there the effects are much more pronounced.^22,88^

**Figure 4:**
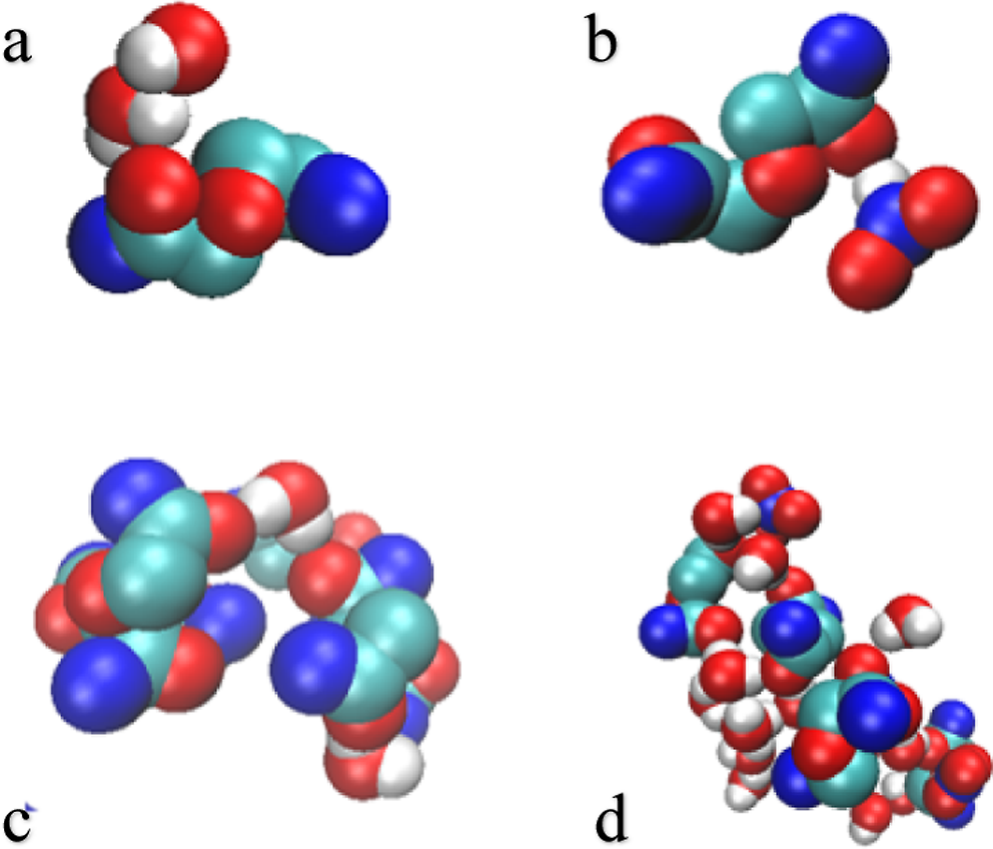
Snapshots of polar molecules interacting with TODGA (hydrocarbon chains hidden for clarity). Red—oxygen, cyan—carbon, blue—nitrogen, white—hydrogen.

There are, however, no clear hydrogen bonding motifs to be observed in the clusters. We show snapshots of how the polar molecules interact with TODGAs in Figure 4 but, unlike, for example, TBP systems where there are clearly defined bridging molecules and strings of molecules connecting TBPs,^22^ this is not so clearly defined in this system. In Figure 4b, we can see hydrogen of nitric acid clearly being attracted to a carbonyl oxygen. In Figure 4c,d, we can see that water also acts as a bridging molecule with its two hydrogen atoms being used to form a hydrogen bonding network. However, it must be stressed that these are not recurring motifs. There is a large variability in these structures and no well-defined molecular strings or bridges. The clusters are irregular and mobile, and while there are correlations between the various polar groups, as evidenced by the radial distribution functions and the co-ordination numbers given in Table 2, the picture is much more that of a rather disorganized polar glue holding the clusters together. For a more detailed analysis of the molecular organization, we refer to Sadhu and Clark.^64^

### Cluster Analysis

**Figure 5:**
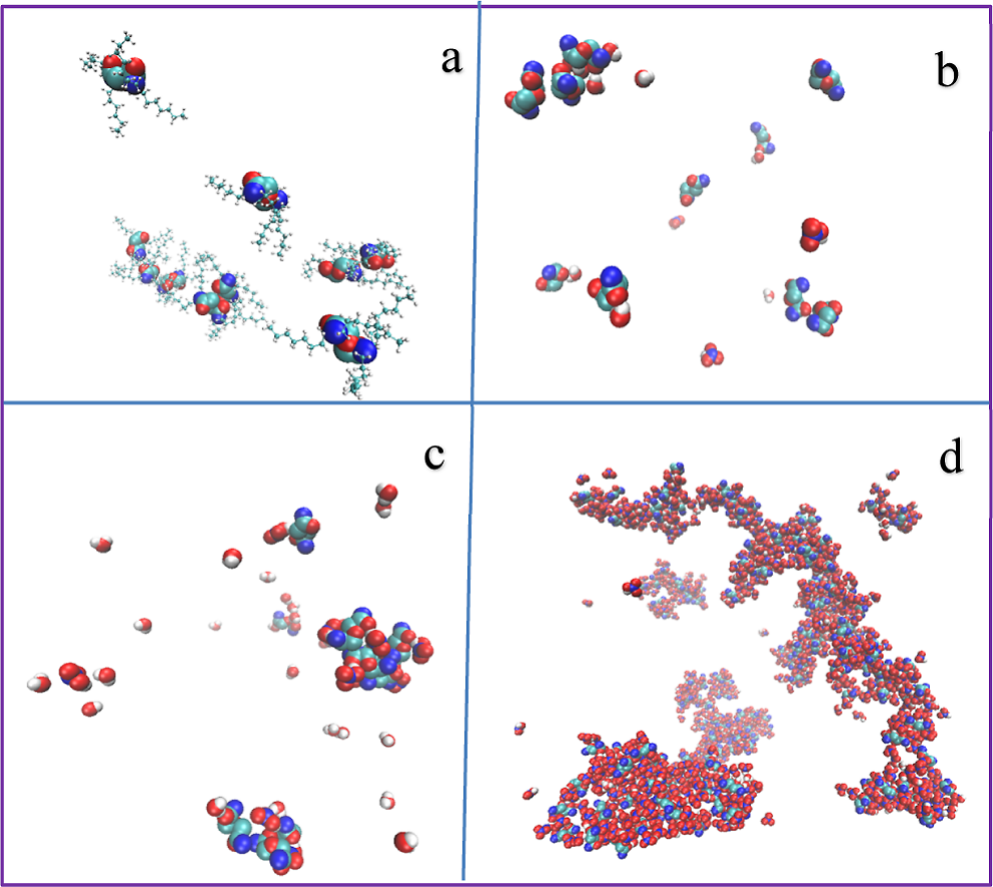
Snapshots of systems with increasing polar contents and TPH hidden for clarity. Red—oxygen, cyan—carbon, blue—nitrogen, white—hydrogen. (a) TODGA-TPH system. (b) System 1. (c) System 3. (d) Third phase.

As shown in the snapshots in Figure 5, the system forms TODGA clusters. At a fixed water/nitric acid ratio, the greater the polar content, the greater the degree of clustering. Further discussion is provided by Sadhu and Clark.^64^

At very high polar contents, the system forms an infinitely connected polar aggregate, which corresponds to the third phase formation. The composition of the simulated system is given in the *Third Phase* column of Table 1. This composition is again not chosen to correspond to systems that have been studied experimentally. It does, however, give an indication of the structure of such phases.

As noted previously, these clusters are irregular in shape and are dynamic in nature. The polar groups are, by and large, in the interior of the aggregate. Such structures are often called reverse micelles in the literature, but it must be noted that the shapes are not simple spheres, rods, or ellipsoids, which are images often conjured up when discussing micelles. Extensive viewing of movies of the simulations show that these aggregates form and break up on a timescale similar to that already reported.

The cluster distribution was analyzed using the hydrogen-bonding criterion discussed earlier, and the results are averages over a 40 ns period. These clusters contain TODGA, nitric acid, and water molecules, and there is a distribution of all three species. To simplify the analysis, we first classify clusters according to the TODGA content alone and later go on to consider the number of polar species, here water and nitric acid, which are also associated with them.

We always observe a distribution of cluster sizes rather than a single cluster size. A simple model for this is the isodesmic model,^38,60,89^ where we assume that the Gibbs energy change of adding a single TODGA molecule to an *n*-cluster, TODGA_*n*_, is independent of *n*. This is tantamount to saying that the chemical equilibrium constant, *K*, for
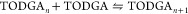
2is independent of *n*. This approximation yields
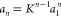
3

**Figure 6:**
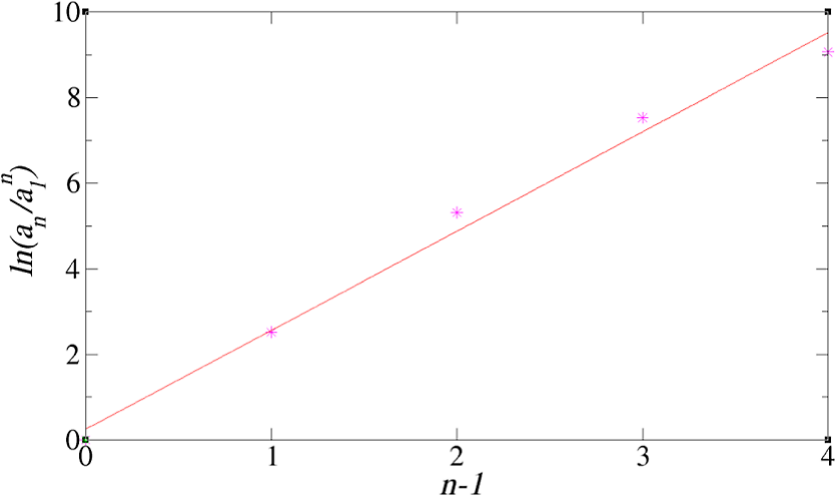
against *n* – 1 for system 3.

Here, *a*_*n*_ is the activity of TODGA_n_. We assume Henry’s law applies, so we have
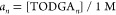
4where we have divided the cluster concentration by the standard concentration of 1 M so as to obtain a dimensionless activity. In Figure 6, we plot  against (*n* – 1) which, if the isodesmic approximation holds, should yield a straight line of slope ln *K*. The graphs indicate that this is a reasonably good approximation and yield equilibrium constants in the range 4–6, the values increasing with increasing polar content of the system. There are, however, discrepancies from the straight line behavior at a large *n*. This is first due to statistical error, for large clusters have smaller concentrations than small ones. The other reason is that eq 3 is only valid for a large system. In our system of 10 TODGA molecules, system size effects will come into play for large clusters. Our estimates for *K* are typically based on cluster sizes up to *n* = 5, where there is good straight line behavior.

These *K* values may be related to the Gibbs energies of aggregation, Δ*G*_agg_, given by
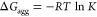
5where *R* is the gas constant and *T* is the absolute temperature. Estimates for *K* and Δ*G*_agg_ are given in Table 3.

**Table 3:** Equilibrium Constants and Gibbs Energies of Aggregation for Systems 1–6^a^

system	*K*	ΔG_agg_/(kJ mol^–1^)
1	6.2 (1.0)	4.52 (0.06)
2	7.2 (1.0)	4.88 (0.06)
3	10.1 (1.0)	5.73 (0.09)
4	8.6 (1.0)	5.33 (0.06)
5	10.8 (1.0)	5.91 (0.06)
6	159 (1.0)	6.85 (0.06)

a Errors are given in brackets.

As noted by Špadina and Bohinc,^1^ the Gibbs energies of aggregation are comparable with thermal energies and the values increase with the amount of polar material present. Many more data points would be needed, however, to establish a quantitative relationship between Δ*G*_agg_ and system composition.

**Figure 7:**
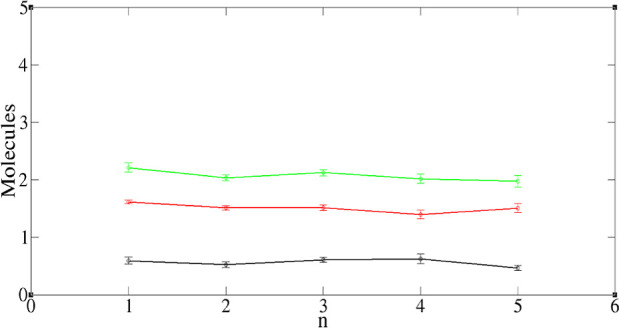
Average number of polar molecules per TODGA vs the TODGA aggregation number, *n*. Black—HNO_3_, red—H_2_O, green—total polar. Data for system 3.

**Figure 8:**
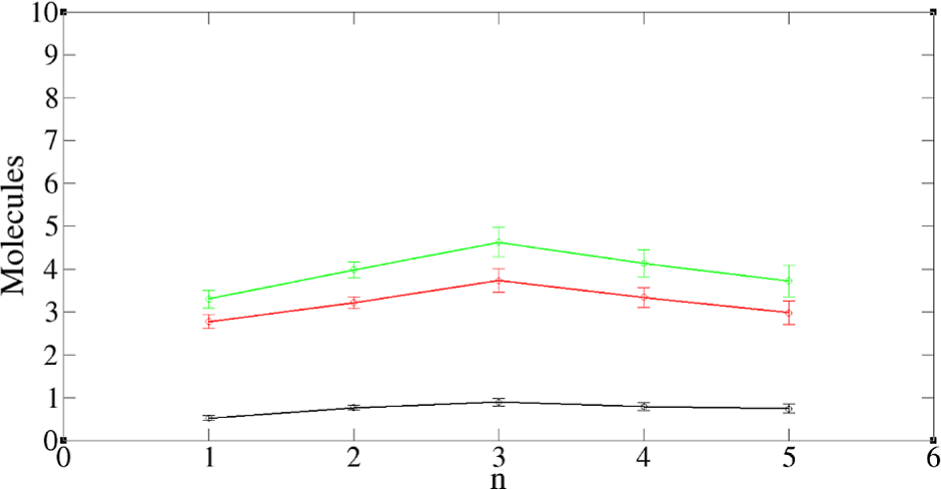
Average number of polar molecules per TODGA vs the TODGA aggregation number, *n*. Black—HNO_3_, red—H_2_O, green—total polar. Data for system 6.

The cluster algorithm can also tell us how many water and nitric acid molecules may be found in an *n*-cluster of TODGA, and again there is a distribution. We provide such data in the Supporting Information. To study how the cluster composition varies with TODGA aggregation number, *n*, we divide the average number of waters and nitric acid molecules in such a cluster by *n*, so as to get the number of polar species per TODGA molecule. In Figures 7 and 8 we plot these quantities against *n* for systems 3 and 6, respectively. For system 3, the lines are approximately horizontal within the statistic error, indicating a roughly constant composition. For system 6, there is also not a great deal of variation, though longer runs on larger systems would be needed to check, for example, whether the maximum at *n* = 3 is statistically significant.

These observations are consistent with the observed isodesmic behavior, which would also suggest that the cluster composition is likely to depend only weakly on aggregation number.

It should be re-iterated, though, that the cluster algorithm used only includes molecules engaged in hydrogen bonding. Thus, there will be water and nitric acid molecules that may well be associated with these clusters but not counted by the algorithm.

### Effects of *n*-Octanol on Cluster Formation

**Figure 9:**
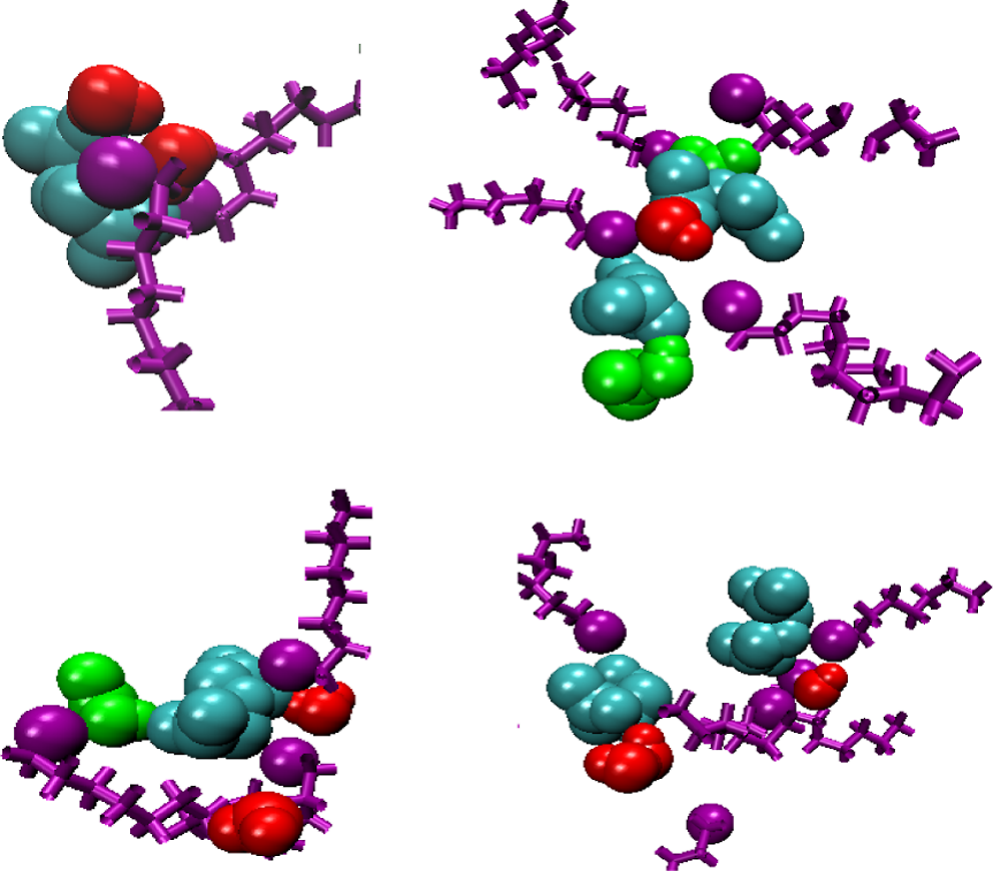
Snapshots of system 1 with added *n*-octanol. Purple—octanol, cyan—TODGA, red—H_2_O, green—HNO_3_.

**Figure 10:**
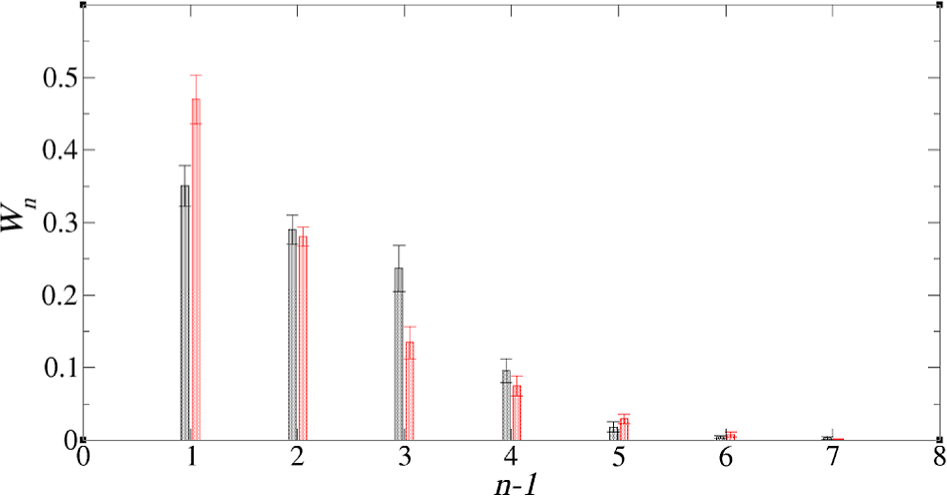
Weighted TODGA distribution in system 3 both without (black) and with (red) *n*-octanol.

As noted in the Introduction*, n*-octanol is also a standard ingredient in the i-SANEX process as it seems to reduce the propensity for forming the unwanted third phase.^55,56^ It also plays a significant role in nitric acid extraction into the organic phase.^11,90^ Abécassis et al. and Lu et al. discuss the various roles that *n*-octanol can play in these systems.^1,55,91^ First, it can act as a co-surfactant and be present in the reverse aggregates. Second, it can act as a co-solvent, affecting the interactions between aggregates. This latter effect is argued to be dominant at high *n*-octanol concentrations.^91^ We have therefore conducted a limited number of simulations for the systems listed earlier but with 20 *n*-octanol molecules added to each. This corresponds to an *n*-octanol concentration of approximately 0.2 M. At this level of concentration, we primarily study the co-surfactant behavior. As may be seen from the snapshots, shown in Figure 9, for the modified system 1 and, more formally, from a co-ordination number analysis, *n*-octanol is incorporated into the clusters and the hydroxyl group interacts with the other polar species present, that is, water, nitric acid, the polar regions of TODGA, and other *n*-octanols. A few *n*-octanols are observed to form small, independent aggregates, typically containing a little water and nitric acid. The full list of co-ordination numbers concerning *n*-octanol are given in the Supporting Information, but, taking system 3 as an example, the co-ordination numbers for the *n*-octanol OH group interacting with the TODGA carbonyl oxygen, water, nitric acid, and a second *n*-octanol OH group are all approximately 0.2. If we look at the TODGA cluster distribution, we see that the degree of clustering is reduced by the addition of *n*-octanol. Taking system 3 as an example, Figure 10 shows how the cluster distribution alters upon the addition of *n*-octanol. The equilibrium constant falls from 10.1 to 7.7. It would appear that *n*-octanol competes with TODGA, to some extent, for binding polar molecules which, in turn, reduces the driving force for TODGA clustering.

The concentrations of *n*-octanol are too small in these cases to observe possible co-solvent effects. Further study is needed on very large systems, however, to investigate the effects of this on aggregate–aggregate interactions.

### Effects of Organic Diluent—*n*-Dodecane vs TPH

**Figure 11:**
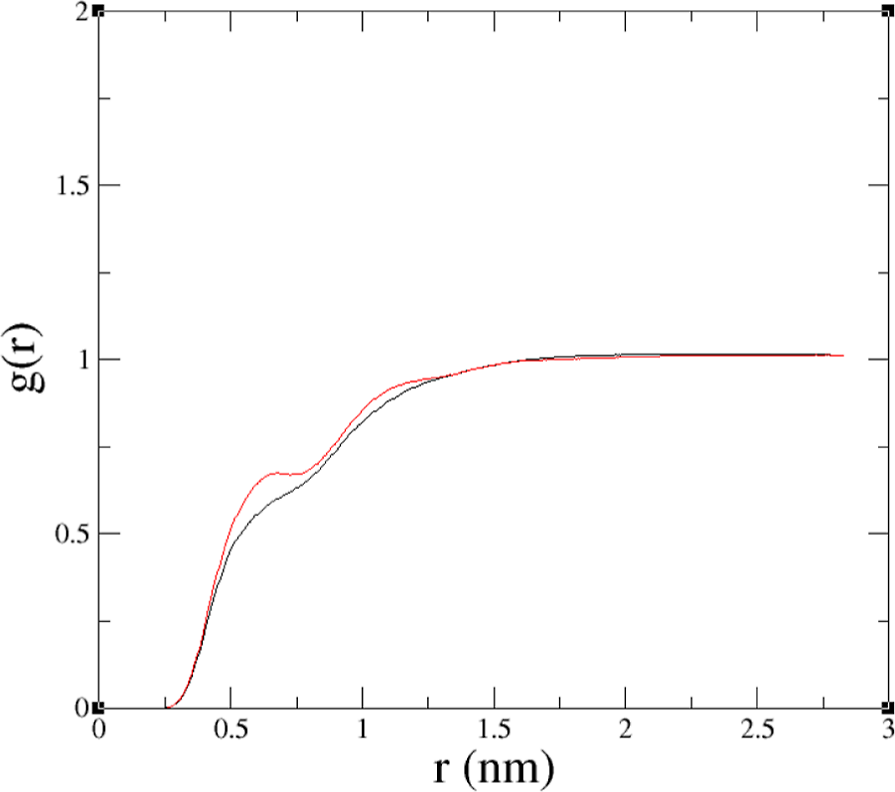
Radial distribution functions in system 3 for the TODGA amide nitrogen/diluent carbon. Red—*n*-dodecane, black—TPH.

It has been observed that changing the organic diluent to less branched alkanes tends to increase the propensity for third phase formation.^27,56,92,93^ While third phase properties are not the focus of this article, this does suggest that a change of diluent could affect clustering in the organic phase containing finite-sized aggregates. We have therefore carried out a simulation where we replaced the branched TPH by linear *n*-dodecane. This was observed to reduce the degree of TODGA aggregation, with, for example, *K* changing from 10.1 to 6.1 in system 3. To help understand why this happens, in Figure 11 we present radial distribution functions of the diluent carbon atoms around the amide nitrogen atom of TODGA. As seen from Figure 1a, this nitrogen is the atom to which the TODGA alkyl chains are attached. The distribution functions show that *n*-dodecane has a small, but significantly greater penetration into the TODGA molecules than does TPH. One might imagine that a straight chain can better worm its way into the interior of a reverse aggregate than can TPH, which has a passing resemblance to barbed wire.^94^ As noted in standard text-books,^23^ in an oil/water/surfactant system, a greater penetration of the oil into the surfactant film leads to a greater curvature of the film toward the water. Our hypothesis is that something similar is happening here. The greater penetration of *n*-dodecane encourages greater curvature of the aggregate, which corresponds to smaller aggregates and a reduced equilibrium constant, *K*.

**Figure 12:**
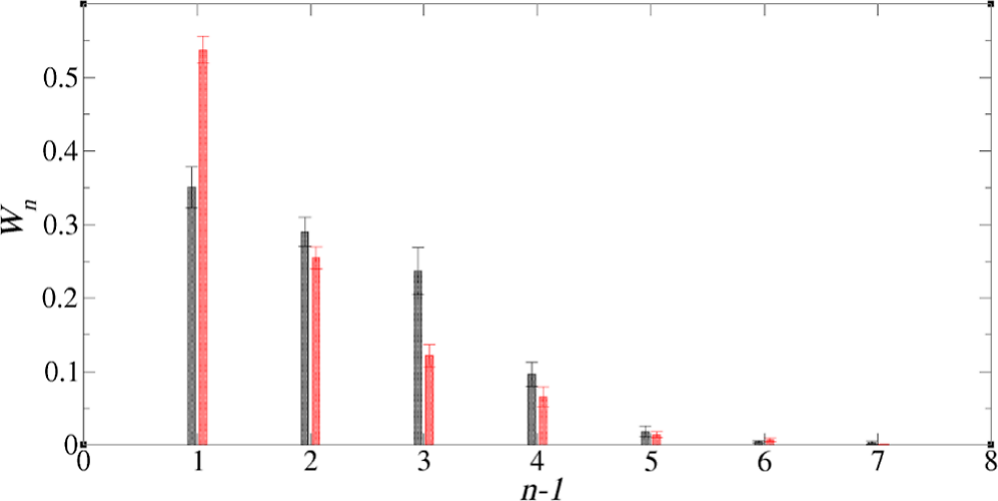
Weighted TODGA distribution in system 3 using TPH (black) and *n*-dodecane (red).

At first sight, this result might seem to be at odds with the fact that third phase formation occurs more readily with *n*-dodecane as the diluent as compared to TPH. If *n*-dodecane tends to reduce aggregation, then one might imagine that its use would also discourage the formation of the extended clusters present in the third phase and thus decrease, rather than increase, the propensity for third phase formation. As is the case with *n*-octanol, however, there are solvent effects which will influence the interactions between aggregates and the change of diluent is likely to affect this. Further studies of the third phase and third phase/organic phase equilibrium will be needed to investigate this question. What the results do show, however, is that there is a definite effect of the diluent on clustering (Figure 12).

## Conclusions

We have conducted a series of molecular dynamics simulations of the organic phase of the metal-free i-SANEX system. The majority of these simulations were for a system of TPH/TODGA/water/nitric acid, though we did more limited studies on the effects of adding *n*-octanol and on the effects of changing the branched TPH diluent to straight chain *n*-dodecane. We studied a range of water/nitric acid compositions to determine the general trends.

The main result is that the TODGA molecules form irregular clusters, with the average cluster size increasing with the polar content of the mixture. This is in qualitative agreement with the results of SANS/SAXS experiments. We also note the distinct roles of nitric acid and water in driving aggregation, and we refer to Sadhu and Clark^64^ for a comprehensive discussion. We studied the bonding that took place within these clusters and found that the carbonyl oxygen was the main site for hydrogen bonding in a TODGA molecule. Typically, nitric acid bound more strongly than water and water more strongly than another TODGA, but all three interactions are present in our simulated structures. These clusters are flexible and mobile, and there is little evidence of dominant hydrogen-bonded bridging structures, though examples of many kinds of linkages may be found. Indeed, it would seem that the interiors of these clusters are somewhat disorganized and that they are held together largely by non-directional polar interactions. A detailed account of the molecular organization within these clusters is given by Sadhu and Clark.^64^

A cluster analysis, based on hydrogen-bonding criteria, predicted a distribution of clusters, which accorded reasonably well with visual inspection of simulation snapshots. The cluster-size distribution appeared to fit an isodesmic model, with the equilibrium constant for aggregation increasing with the polar content. Typically, this equilibrium constant, based on molar concentrations, was in the range of 4–6.

The addition of *n*-octanol was observed to decrease the amount of TODGA clustering, reducing the aggregation equilibrium constant. A structural analysis showed that at the relatively low concentrations studied, the majority of the *n*-octanol molecules were incorporated into the TODGA clusters. Our hypothesis is that competition between *n*-octanol and TODGA for favorable polar interactions is responsible for this decrease.

Replacing the branched TPH diluent with straight chain *n*-dodecane also had the effect of decreasing the degree of clustering. We argue that this is because *n*-dodecane penetrates a reverse aggregate to a greater extent than TPH, probably due to the fact that TPH has a branched structure and so there is additional steric hindrance. Such penetration is known to increase the curvature of a surfactant film in an oil/water system and, as a small aggregate is move curved than a large one, this effect is expected to reduce aggregate size.

It would, of course, be useful if simulation work, such as this, could be used to inform the flow-sheeting modeling that is used at the process level. In these flow-sheet models, it is common practice to represent the organic and aqueous phases as containing well-defined clusters, with a specific chemical formula, and thereby model cluster–cluster equilibrium. Simulation, however, suggests a much more disordered state of affairs, with loose aggregates comprising a wide range of possible compositions. Process modeling studies also hint at this,^11^ with the model requiring a range of nitric acid/TODGA complexes. How best to represent such a system in practical flow-sheet terms is far from obvious, but if the isodesmic behavior reported here is general to many systems, this may prove to be a possible way of incorporating aggregation effects into a practical model.

## Supporting Information Available

Nitric acid parameters; parameters of the polar part of TODGA; pair correlation functions of system 3 for TODGA–TODGA, TODGA-HNO_3_, TODGA-H_2_O, HNO_3_–HNO_3_, HNO_3_–H_2_O, and H_2_O–H_2_O; plot of ln(*a*_n_/(*a*_1_^*n*^)) against n-1 for systems 1–6; average number of water and nitric acid molecules in a cluster of n TODGAs; data for systems 3 and 6 along with error estimates; plot of ln(*a*_n_/(*a*_1_^*n*^)) against n-1 for *n*-octanol-containing system 3; pair correlation function of octanol system 3; octanol–octanol, octanol-TODGA, octanol-H_2_O, and octanol-HNO_3_; plot of ln(*a*_n_/(*a*_1_^*n*^)) against n-1 for system 3 with TPH replaced with dodecane; and plot of the average number of TODGA in clusters against time for system 6 (PDF)
