# Supplementary material for: Molecular Dynamics Study of the Aggregation Behavior of N,N,N′,N′-Tetraoctyl Diglycolamide
Source: J Phys Chem B. 2022 Aug 17;126(33):6290–300. doi: 10.1021/acs.jpcb.2c02198 (PMC9421649; doi:10.1021/acs.jpcb.2c02198)
Supplement: Supplementary file 1 — jp2c02198_si_001.pdf [file jp2c02198_si_001.pdf]

Supporting Information

A Molecular Dynamics Study of the Aggregation Behaviour of N,N,N′,N′-Tetraoctyl Diglycolamide (TODGA)

AUTHOR NAMES: Daniel Massey,[֍ϟ] Andrew Masters,[֍ϟ] Jonathan Macdonald-Taylor,[ǂ] David Woodhead,[⁜] Robin Taylor[⁜]

[֍]Department of Chemical Engineering, The University of Manchester, Oxford Road, Manchester M13 9PL, U.K.

[ǂ]National Nuclear Laboratory, 5th Floor Chadwick House, Warrington Road, Birchwood Park, Warrington, WA3 6AE, United Kingdom

[⁜]National Nuclear Laboratory, Central Laboratory, Sellafield, Seascale, CA20 1PG, United Kingdom

Corresponding Author

[ϟ]E-mail: daniel.massey@manchester.ac.uk

[ϟ]E-mail: andrew.masters@manchester.ac.uk

1. Nitric acid forcefield

Type	Atom type	charge	sigma (nm)	epsilon(kJ/mol)
opls_760	NO	0.968	3.25000e-01	5.02080e-01
opls_761	ON	(-0.408, -0.497)	2.96000e-01	7.11280e-01
opls_023	OH	-0.526	3.07000e-01	7.11280e-01
opls_024	HO	0.463	0.00000e+00	0.00000e+00

Table S1 - Nitric acid parameters.

Atom	charge
O1	-0.688
C1	0.384
C2	0.949
O2	-0.744
N1	-0.821
C19	0.384
C20	0.949
O3	-0.744
N2	-0.821

Table S2 - Parameters of polar part of TODGA.

2. Pair correlation functions

We present here a set of pair correlation functions for key pairs of atoms. As well as providing information about the liquid structure, they also give the values for r_{min} used in the co-ordination number analysis.

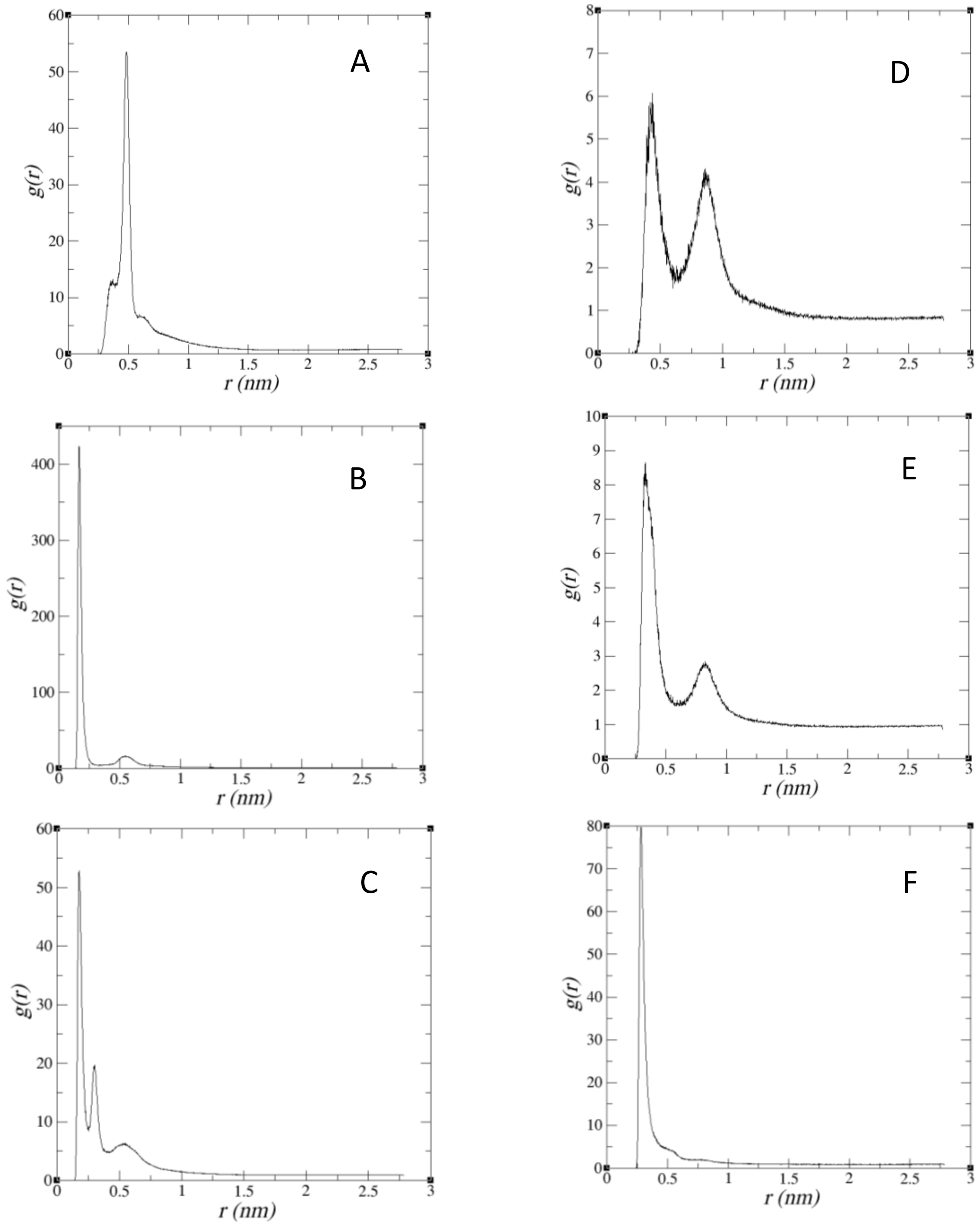

Figure S1 – Pair correlation functions of system 3 for TODGA-TODGA (A), TODGA-HNO_3 (B), TODGA-H_2O (C), HNO_3-HNO_3 (D), HNO_3-H_2O (E), and H_2O-H_2O (F).

3. Determination of the equilibrium constant for aggregation

We present plots of $\ln(a_n/(a_1^n))$ against n-1 for system 1-6 to indicate the quality of the isodesmic equilibrium assumption.

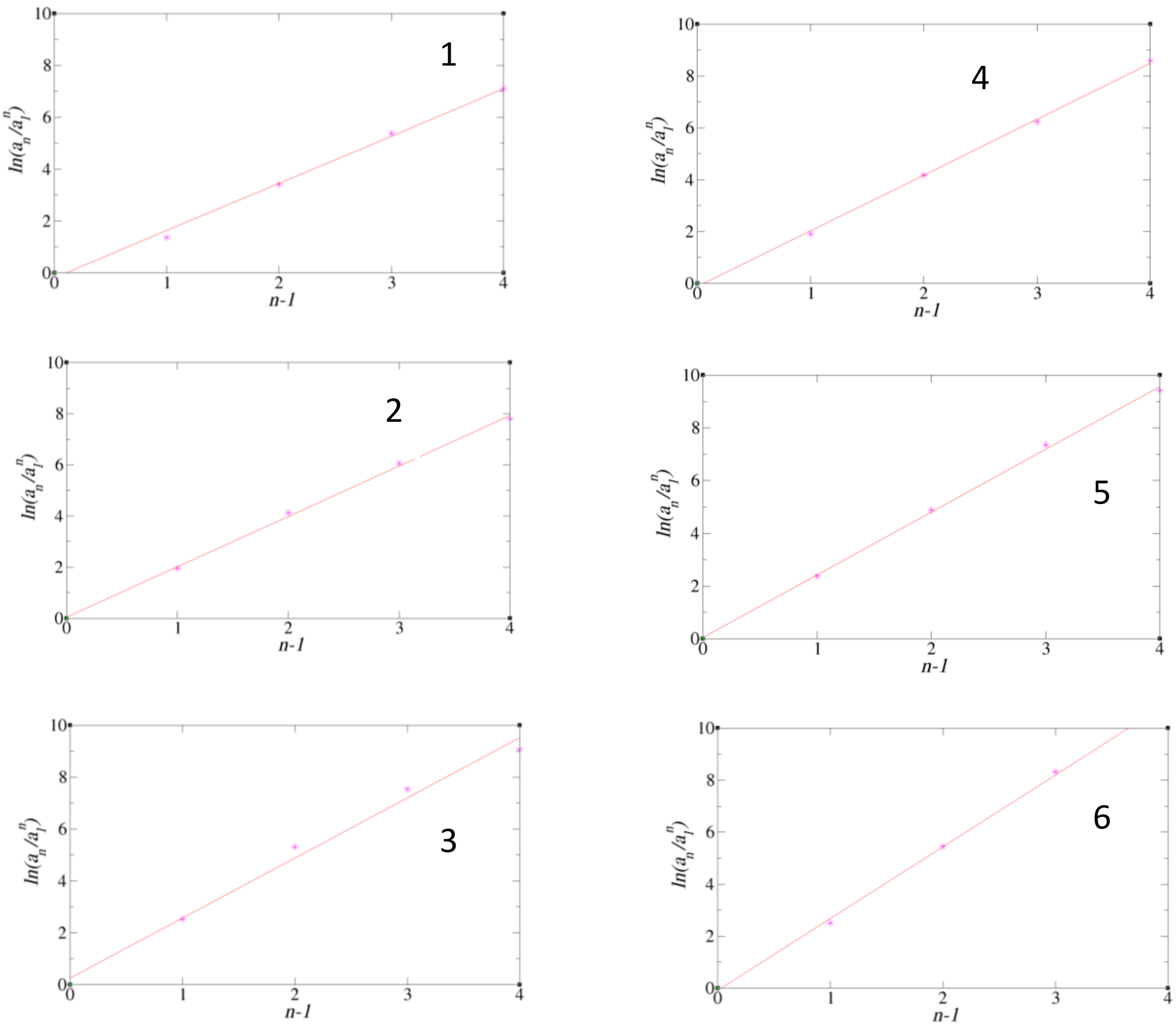

Figure S2 - Plot of $\ln(a_n/(a_1^n))$ against n-1 for system 1-6.

4. Composition of the clusters in Systems 3 and 6

We provide data for the average number of nitric acid and water molecules in a cluster of *n* TODGA molecules, as observed in Systems 3 and 6.

TODGA	System 3		System 6	
	HNO_3	H_2O	HNO_3	H_2O
1	0.596 (0.06)	1.62 (0.03)	0.527 (0.06)	2.78 (0.16)
2	1.05 (0.10)	3.03 (0.08)	1.54 (0.10)	6.44 (0.28)
3	1.83 (0.13)	4.55 (0.15)	2.69 (0.25)	11.2 (0.81)
4	2.50 (0.35)	5.59 (0.31)	3.18 (0.37)	13.4 (0.94)
5	2.35 (0.20)	7.56 (0.39)	3.74 (0.52)	14.9 (1.39)
6	3.53 (0.59)	10.2 (1.3)	5.22 (0.52)	23.0 (0.84)
7	3.65 (0.71)	9.88 (1.9)	6.00 (0.31)	24.7 (1.61)

Table S3- Average number of water and nitric acid molecules in a cluster of n TODGAs. Data are given for systems 3 and 6. Error estimates are in brackets.

5. Determination of the equilibrium constant for aggregation in the presence of *n*-octanol

We present plots of ln($a_n/(a_1^n)$) against *n*-1 for system the octanol containing system to indicate the quality of the isodesmic equilibrium assumption.

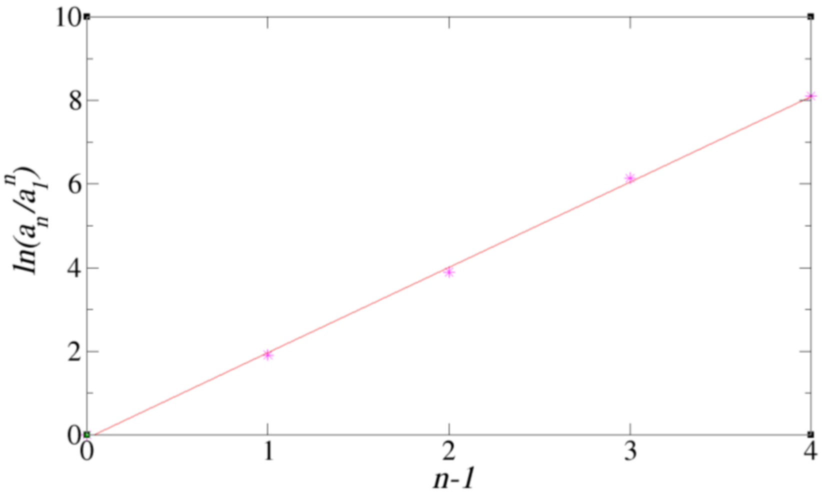

Figure S3 - Plot of ln($a_n/(a_1^n)$) against n-1 for the n-octanol containing system 3.

6. Pair correlation functions for the *n*-octanol system

We present here a set of pair correlation functions between the hydroxyl group on *n*-octanols and other key atoms in the system. As well as providing information about the liquid structure, they also give the values for r_{min} used in the co-ordination number analysis.

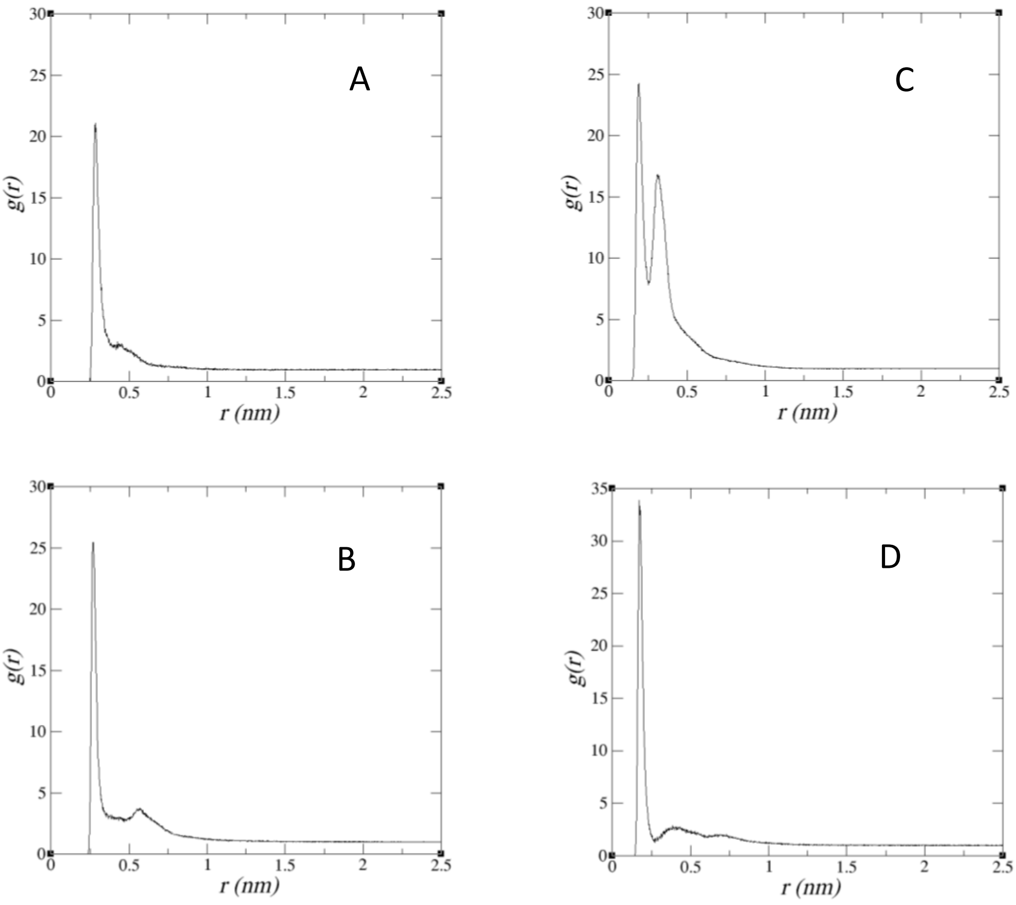

Figure S4 – Pair correlation function of octanol system 3. Octanol-octanol (A), octanol-TODGA (B), octanol-H_2O (C) and octanol-HNO_3 (D).

7. Determination of the equilibrium constant for aggregation with *n*-dodecane

We present plots of ln($a_n/(a_1^n)$) against *n*-1 for system 3 where n-dodecane replaces TPH. This helps indicate the quality of the isodesmic equilibrium assumption.

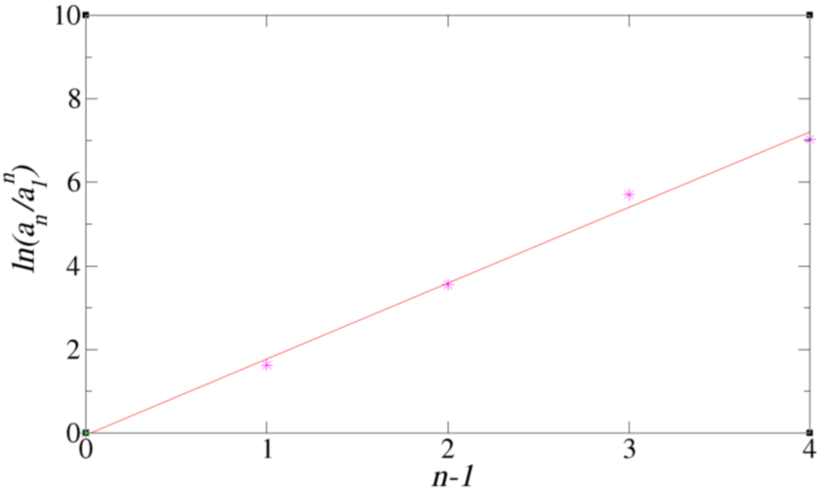

Figure S5 - Plot of ln($a_n/(a_1^n)$) against n-1 for system 3 with TPH replaced with dodecane.

8. Distribution of TODGA clusters over time

We present an illustrative plot, showing the distribution of TODGA clusters over time for System 6, which is the system with the greatest water and nitric acid content. As may be seen, there is no indication of increased clustering with time and thus no indication of phase separation. Similar plots are found for all systems studied.

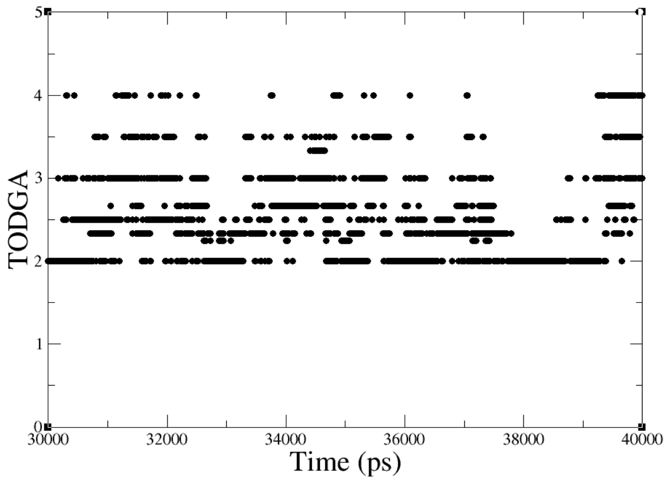

Figure S6 –Plot of the average number of TODGA in clusters against time for system 6.
